# Head and neck lymphoedema service provision in the UK: a survey of practice

**DOI:** 10.1007/s00520-025-10107-6

**Published:** 2025-11-27

**Authors:** Alison J. Smith, Mary Cherry, Joanne Patterson

**Affiliations:** 1 Faculty of Health & Life Sciences, University of Liverpool, Liverpool, UK; 2 University Hospitals Coventry & Warwickshire, Coventry, UK

**Keywords:** Head and neck cancer (HNC), Head and neck lymphoedema (HNL), Service provision, UK survey of practice

## Abstract

**Purpose:**

As the incidence of head and neck cancer (HNC) is increasing, patients are living for longer with late effects of HNC treatment, one of which is head and neck lymphoedema (HNL). Whilst HNL has been hugely under-reported and under-treated, recent studies have identified up to 90% of patients who have HNC treatment can develop HNL. This can be as devastating as the cancer diagnosis and treatment itself, but last many years longer, impacting on quality of life (QOL), swallowing function, nutrition, hydration, social isolation, depression and appearance. It is important to determine availability of services and treatments for patients with HNL and understand the differences in health care services provided in these settings to identify gaps in provision.

**Methods:**

A two-part Qualtrics questionnaire was distributed to health professionals involved in the HNC Multi-Disciplinary Team via social media platforms, HNC-related organisation websites/accounts and a UK HNC Support Group.

**Results:**

The survey received 169 responses, 134 of which were analysed as the final data set once test and incomplete entries were eliminated. Participant narratives were described using content analysis and descriptive statistics.

**Conclusion:**

This survey suggests a large proportion of HNC patients are not being referred to services compared with the documented incidence of HNL in this patient group after treatment. This disparity in assessing and treating HNL across the UK is consistent with available published literature. Barriers to referring and accessing services are multi-factorial for referrers and patients alike.

**Supplementary Information:**

The online version contains supplementary material available at 10.1007/s00520-025-10107-6.

## Background

 Head and neck lymphoedema (HNL) is a chronic condition affecting up to 90% of patients treated for head and neck cancer (HNC) [1, 2]. HNC treatments (surgery, radiotherapy ± chemotherapy) have substantial impact on lymphatic drainage, resulting in an accumulation of protein-rich fluid, fibrosis and fat in the interstitial tissues [3, 4]. Lymphoedema after HNC is more pronounced than in other cancer groups due to the complex lymphatic network in this anatomical area, and the use of multi-modal treatments [1, 3]. HNL can increase in severity over time leading to fibro-fatty scarring, highlighting the need for early intervention [3]. External HNL causes swelling of the face, neck, shoulders, lips and eyelids, whereas internal HNL affects the tongue, cheeks, oro-pharynx and larynx. Consequently, this results in altered appearance, as well as functional problems with speech, breathing and swallowing [2]. Whilst HNL assessments often include physical and functional domains, often emotional and psychosocial impacts are underrepresented [5]. Previous qualitative research investigating the psychosocial impact of HNL highlighted themes of avoiding social interactions, adaption or avoidance of returning to work and a poorer quality of life (QOL) relating to altered appearance and financial burden [4]. Qualitative interviews investigating patients understanding, perceptions and experiences of HNL management identified key barriers diminishing patients’ motivation and competency in HNL self-management, including lack of understanding, appropriate and timely tailored education and HNL not being a patient priority [6]. The timing of education and HNL input was also raised by patients in this study with most participants reporting very little knowledge surrounding what HNL was at the end of treatment, or how to manage it. There are no studies specifically that explore how patients currently learn about HNL. This study highlights the need for HNC services to raise awareness of HNL and its chronicity, to improve the biopsychosocial burden placed on patients and promote motivation for self-management [6]. In addition, as a result of the burden of multiple treatment appointments, the role of self-management has been promoted within HNL management, suggesting a shift away from more traditional professional-led approach [6].

The evidence base for effective HNL assessment and interventions is limited, with no consensus on the optimal management of this condition [7, 8]. In a fast-developing field of practice, investigating the efficacy of interventions without agreed, standardised HNL assessments is a challenge. Information on HNL-specific services in the United Kingdom (UK) are unknown with no single referral process or standardised clinical pathway [9]. Internationally, access to general lymphoedema services appears variable, under-resourced, disjointed and randomly located [10–14]. General lymphoedema services are led by a range of health professionals such as specialist nurses, physiotherapists, occupational therapists, massage or manual therapists and speech and language therapists [10–14]. More detailed information regarding what services provide assessment and treatment of HNL across the UK and what methods they use is needed to identify potential gaps in provision. This survey of practice thus aims to determine availability of services and treatments for patients with HNL following surgery, chemotherapy and/or radiotherapy treatment for HNC in the UK.

## Methods

### Study design

This was a cross-sectional, self-report online survey. Ethical approval was obtained from the Institute of Population Health Research Ethics Committee at the University of Liverpool (Ref: 13434).

### Survey development

Survey questions were informed by a scoping review on the assessment and management of HNL after HNC and developed by the research team who bring expertise in Speech and Language Therapy and Clinical Psychology [14]. To ensure that the survey items appropriately reflected the construct under investigation, face validity was assessed prior to data collection. The survey was refined following a pilot with four specialist speech and language therapists and one HNC specialist nurse who provided feedback on the structure and design. Based on their feedback, minor revisions were made to improve wording and remove any ambiguities. Sections of ‘referrer’ and ‘treating clinician’ were separated, and suitability of the questions in terms of ‘management’ vs ‘treatment’ wording was changed.

The final survey comprised two parts to establish a current picture of lymphoedema management across the UK. Part A contained 12 questions capturing demographic characteristics of those responsible for making referrals, what influences those decisions, their caseload mix, what regional service provision is available to them in the UK and possible barriers to accessing these services. Part B comprised 14 questions to clinicians who provide HNL management and the training they have received. Questions were designed to establish current methods of assessment, interventions, contra-indications and equipment used. A combination of Likert and open ended free-text responses was used to capture confidence ratings factors and decision-making when treating patients. The survey was uploaded onto the secure online tool Qualtrics. An information sheet was provided digitally and consent was requested on the first web page of the online survey.

### Participants

Inclusion criteria were any healthcare professionals, working within a multi-disciplinary team (MDT) for HNC patients within the UK. Exclusion criteria included any healthcare professionals not working within HNC care.

### Procedure

Convenience sampling was used to distribute the survey link. A two-part Qualtrics questionnaire was distributed to health professionals involved in HNC MDTs via social media platforms, HNC-related organisation websites/accounts and a UK HNC Support Group. ‘Clinical Excellence Networks’ which are part of the professional body of the Royal College of Speech & Language Therapists, social media links (Twitter/X, Instagram, Facebook) and relevant organisations for distribution to their members (British Lymphology Society, Liverpool Head and Neck Cancer Centre, Macmillan Cancer Support, Myton Hospice) and THE SWALLOWS (a UK head and neck cancer support group). Attempts at obtaining a further reach of distribution to physiotherapists and occupational therapy networks was limited by costs to advertise the link and the length of time required to distribute exceeding the survey deadline to complete.

### Data collection

The web survey link contained an information sheet detailing aims of the study and a confidentiality statement with a QR code. The link was disseminated three times, across three weeks, on different days in the week, to act as a prompt to complete and to achieve maximal response rate, closing on 31 March 2024.

### Data analysis

Data storage was compliant with General Data Protection Regulations. Survey data were extracted and entered into Microsoft® Excel® for Microsoft 365 MSO (Version 2402 Build 16.0.17328.20124). Entries were screened for any errors or data entry anomalies prior to data cleaning by eliminating test entries, repetitions or if participants had not agreed to the disclaimers which prevented the survey opening. Data entries that were over 50% incomplete were discarded.

All open-text responses were exported into Excel for analysis after de-identification. A conventional content analysis approach was used to inductively derive categories from open-ended, free text survey responses [15]. Initial codes were generated inductively. Data was sub-categorised into codes and central organising concepts which were discussed and refined in discussion with the research team to develop themes of symptoms, barriers to treatment and respondent knowledge and skills. Responses were coded line-by-line with sentence or key word level, e.g. ‘appearance’ or ‘swallow’, when considering functional symptoms that may prompt a referral. The codebook was iteratively refined through discussion. Codes were grouped into broader themes representing patterns in clinical practice. Themes were reviewed by the research team to ensure representativeness. Descriptive statistics including percentages and frequency distributions were used to summarise numerical data.

As researchers with backgrounds in clinical practice, we acknowledge that our professional training, clinical roles, and prior experiences in the NHS healthcare system shape the perspectives we bring to analysis of this study. Alison Smith is a practicing lymphoedema clinician who may hold assumptions about standard care pathways, which could influence how survey responses are interpreted. We recognise these positions of expertise, and we have sought to mitigate bias by using collaborative interpretation across team members with varied professional experiences.

## Results

Of the 169 responses were received, 35 were incomplete and removed, leaving 134 responses available for analysis.

## Part A

### Respondent demographics

A range of professionals participated. Table 1 provides an overview of respondents’ demographic information.

**Table 1:** Demographic and professional profile of respondents completing the survey

Responders (*n* = 134)		
	Frequency	
**Characteristics**	*N*	%
Dietitian	3	2%
HNC CNS	7	5%
HNC Oncologist	3	2%
HNC Radiographer	2	1%
Lymphoedema Nurse	46	34%
OT	3	2%
PT	17	13%
SLT	43	32%
Other	9	7%
Missing	1	1%
**Setting**		
Hospice	12	9%
Inpatient	4	3%
IP/OP	54	40%
IP/OP/Hospice	5	4%
OP	48	36%
OP/Hospice	4	3%
Missing	7	5%
**Geographical area**		
East England	3	2%
London	18	13%
Midlands	36	27%
North East & Yorkshire	18	13%
North West	8	6%
Northern Ireland	10	7%
Scotland	7	5%
South East England	12	9%
South West England	6	4%
Wales	11	8%
Missing	5	4%

### Referrals to services

Approximately half of respondents making referrals had access to HNL services based in community NHS clinics. Under half (43%) of respondents had access to in-house services via an inpatient (11%) or outpatient (32%) setting. Only 4% of respondents were unable to access any form of HNL service.

Mean confidence of respondents recognising internal and external lymphoedema in order to make a referral was 5.9 and 8.3 out of 10, respectively (Table 2). Most (81%) of respondents were able to refer to lymphoedema services without prior consultant approval.

**Table 2:**
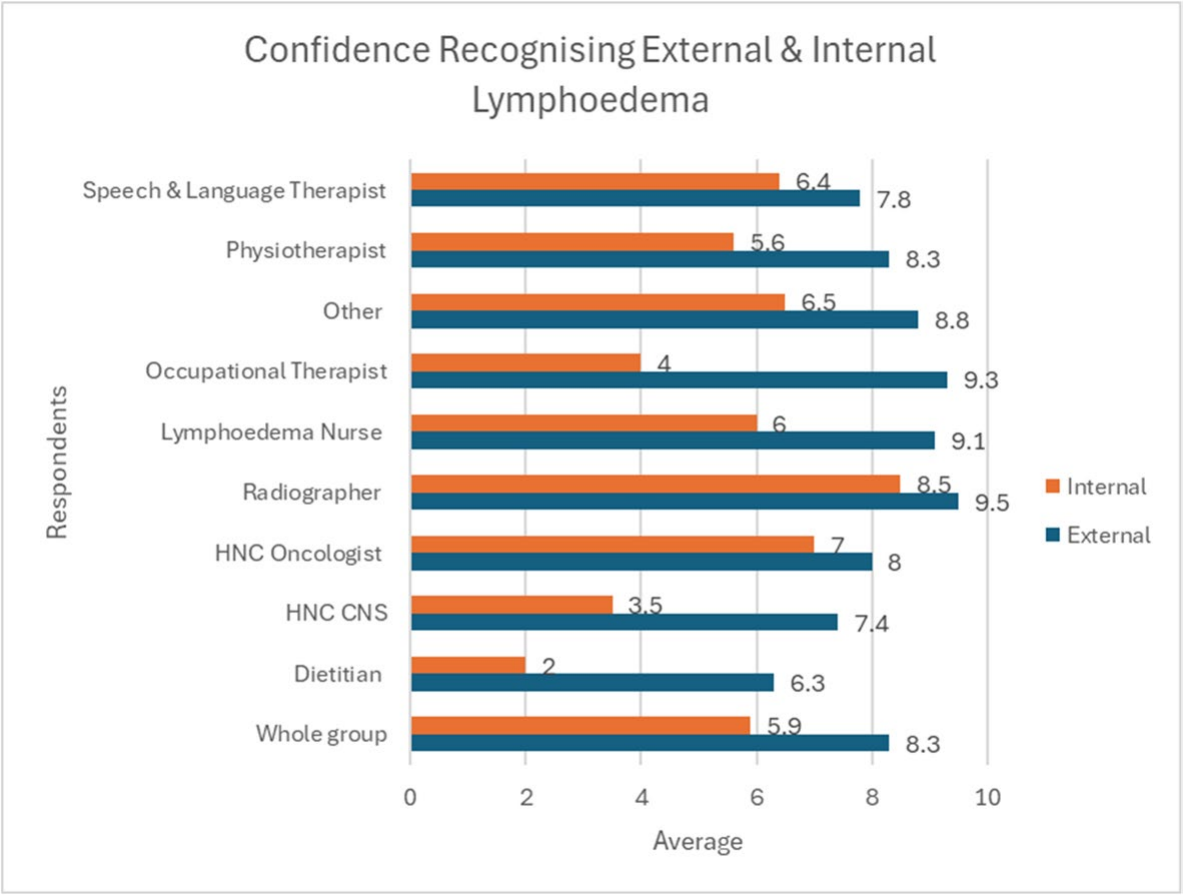
Mean confidence of respondents recognising internal and external lymphoedema

Factors influencing clinicians’ decision of whether patients would benefit from a referral to a lymphoedema service were collated from free text answers, grouped into central organising concepts and developed into themes (Table 3); the physical and psychosocial impact on patients, staff expertise and availability of services.

**Table 3:** Factors influencing clinicians’ decision

Theme	Central organising concepts	Codes	
Impact on the patient	Functional	Presence of dysphagia/dysarthria (13)	“We use symptoms eg dysphagia to determine need for onward referral to specialist lymphoedema service”
		Impact of HNL on dysphagia (17)	“I consider if there is external lymphoedema likely there is internal which will impact their swallow”
		Neck, shoulders & face range of motion (16)	“issues with neck movement”
		Pain or discomfort (16)	“Tightness, discomfort, pain”
	Psychological/psychosocial	Impact on activities daily living (3)	“I consider how much it is impacting their day to day life”
		Quality of life (26)	“If they have swelling that is causing discomfort physically or mentally to them”
		Distress (2)	“patients distress at the HNL”
		Patient reported concerns (10)	“whether it’s concerning to the patient”
		Appearance/cosmesis (16)	“Patients usually talk about appearance and how they have developed a ‘turkey neck’.”
	Profile of HNL symptoms	Visually overt (26)	“How visible is the lymphoedema (either externally or seen internally on FNE)”
		Severity/continuum (25)	“Severity of oedema. Area of oedema”
		Texture/firmness (6)	“if pitting present. If fibrosis present”
		Fibrosis/scars (9)	“Skin and tissue condition – scarring and mobility of the tissues”
Staff knowledge & expertise	Decision-making for referrals	Process/consideration of referral (12)	“Factors to consider are cancer stage, history of treatment and level of side effects, past medical history, airways, level of pain, patient’s level of understanding and body awareness, patient engagement, aims and what is realistic to achieve”
		Objective assessment factors (7)	“Raised lymphscanner readings”
		Subjective assessment factors (7)	“What the tissues feel and look like”
		Recognition of patient reported symptoms (9)	“Patient self reported swallowing issues associated problems such as saliva production, tongue mobility, tissues/fibrosis, scarring, range of movement/infection/skin integrity”
		Patient engagement with self-management (17)	“Ability of patient to manage swelling. Whether the patient would want any input”
		Need for early intervention (4)	“Also knowing that the earlier it is treated the better to reduce it solidifying”
Availability of services	Timing of referrals	Which clinician refers (8)	“I only see patients who are referred by the head and neck clinic or GP so someone else has already made the decision that patient might benefit from treatment. The referrals are appropriate, but I don’t know if we are missing patients who have not been referred”
	Impact of self-management	Provision of exercises/self-lymphatic drainage (4)	“Everyone referred to our Lymphoedema service is prescribed with appropriate lymphoedema treatment, even if they are only at risk of suffering from lymphoedema. Depending on the presentation and patients needs, it will imply patient education on self-management that can include: head and neck exercises and good posture, skin care, compression, scar/fascia management, kinesio-tape, self-lymphatic drainage. If appropriate, the patient will be offered to undergo intensive treatment”
		Information giving/education (4)	“Even if just being given explanation/clarification on lymphoedema and what causes what they perceive in their head and neck region is part of lymphoedema management”

## Part B

### Respondent knowledge and skills

Half of respondents provided HNL treatment, comprising physiotherapists (20%), lymphoedema nurses (44%), speech and language therapists (5%), service leads (2%), independent practitioner/therapists (1%) and (1%) respectively for CNS, health and social care worker, HNC radiographer and occupational therapist. Forty respondents (57%) were formally certified lymphoedema therapists that had undertaken 135 hours of training as the highest source for lymphoedema education, with or without additional competency frameworks or uncertified training courses. There were four respondents that had received peer learning only and two respondents who have received no training at all.

Table 4 displays the characteristics of services from where participants were based. The majority provided out-patient HNL services postoperatively (78%) and post chemo-radiation (74%). Two-thirds of respondents saw patients for palliative and late effects management. Over 80% of participants providing services offered assessment, treatment, self-management programmes and reviews. Most (82%) respondents reported offering as many follow-up appointments as patients required. Over half (57%) offered a set number of follow-up appointments (between 3 and 10), for up to 10 years post-treatment.

**Table 4:** Service provision provider demographics and service characteristics

Responders (*N* = 69)					
**Who do you accept referrals from?**	*N*	%	**What do you offer in terms of input in your service?**		
Oncologist	68	98%	Initial Ax	60	86%
H&N surgeon	68	98%	Txt delivered by clinician	59	85%
Patient self-referral	22	31%	Txt taught for home programme self	56	81%
SLT	65	94%	Direct follow ups	57	82%
OT	61	88%	Missing	1	1%
PT	67	97%			
H&N CNS	66	95%			
Palliative CNS	49	71%			
Other	36	52%			
* GP, DN, Community Nurse, Practice Nurse, Dentist, Dermatologist, Late Effects, Dietitian, Radiographer, Ward Staff, Plastics					
**What training have you received relating to HNL?**			**At what point in the HNC pathway do you see patients?**		
120 h full certification	40	57%	Pre-chemo & radiation	17	24%
120 h full certification AND In-house competency programme	14	20%	During chemo or radiation	21	30%
Purely in-house learning with no other training	6	8%	Post chemo and radiation	51	74%
120 h full certification AND other external course	12	17%	Late effects	46	66%
Other external courses only (not 120 h certified)	15	21%	Palliative	46	66%
Peer learning only	4	5%	Post op IP	10	14%
Peer learning AND 120 h full certification, OR other external courses (not 120 h certified)	30	43%	Post op OP	54	78%
No training at all	2	2%	Pre-surgery education and preventative	7	10%
			Missing	4	

### Assessment in practice

Assessment methods and devices used are outlined in Table 5. Almost half (40%) of respondents used standardised assessment tools; 11% used a formal rating scale for lymphoedema volume and texture; 31% included a patient reported outcome. Only 2% used an objective skin firmness measure.

**Table 5:** Service provision assessment and intervention

Responders (*N* = 69)					
**Do you use any standardised assessment tools?**			**Treatments offered**		
Yes	28	40%	MLD by clinician	55	79%
No	30	43%	SLD	68	98%
			Skincare	67	97%
**Do you use formal rating scale for volume and texture of HNL**			Neck exercises	67	97%
Yes*	8	11%	Compression	63	91%
No	50	72%	Facial exercises	57	82%
* MDACC, Patterson Internal Lymphoedema Scale, ISL, 5 s score, ILF staging			Hereford collar	45	65%
			Intra-oral drainage clinician led	12	17%
			Intra-oral drainage SLD	25	36%
**Do you use any QOL questionnaires for HNL**			Swallow exercises	25	36%
Yes*	22	31%	Kinesiotape	49	71%
No	37	53%	Other (please state)	13	18%
*LymQol, Welsh LYMfunc, Psychological ax form, Lymprom, own in house developed PROM, Lymphoedema impact score			Low level laser	11	15%
			PBM Thor	17	24%
			PBM CareMIN	1	1%
			Pneumatic compression device	1	1%

### Treatment in practice

Treatments offered are summarised in Table 5. Self-management programmes and in-person treatment were common (81% and 85%, respectively). Complete decongestive therapy (CDT) was the most frequent HNL treatment offered, comprising skincare (79%), neck exercises (97%), compression (92%) and skincare (97%), in addition to Kinesiotape (71%). Intra-oral drainage was less common (17% provided by clinicians, 36% self-directed).

Contraindications to treatment are outlined in Table 6. Active disease, recurrence or fungating tumours (42%) were the most commonly cited reasons. Other less common reasons were cognitive impairment, suspicion of recurrence, fistula, no consultant approval, medications, fibrosis, unstable cardiac failure and superior vena cava obstruction.

**Table 6:** Contraindications to treatment

Contraindications		
Fistula	1	1%
Carotid/blood vessel disease/arteriosclerosis/CVA history	9	13%
Time post txt/during txt	9	13%
Progression of lymphoedema	1	1%
Active disease/recurrence/fungating tumours	29	42%
Wounds, lymphorhea	7	10%
Poor tolerance/compliance/no response to txt	4	5%
Medically unstable/fatigue	5	7%
Infection	8	11%
Skin reaction/integrity	4	5%
Cognitive impairment	1	1%
Airway/breathing/tracheostomy	4	5%
Pain	6	8%
Cellulitis	4	5%
No Consultant approval	1	1%
Medications	1	1%
Fibrosis & setting expectations	1	1%
Unstable cardiac failure	1	1%
SVCO	1	1%

### Barriers to patients receiving treatment

Clinician skills and knowledge, service capacity restrictions, stage of cancer pathway and patient acceptability were identified in open text responses as potential barriers to patients accessing services. Multiple barriers to access were categorised into the service level (*waiting lists*,* understanding the need for early intervention*,* lack of services/capacity*), the referral level (*profile/severity of HNL*,* clinician skills in recognising HNL*,* contraindications/cautions*,* cancer status/timing of input*) and the patient level (*patient motivation*,* distress*,* quality of life*,* transport and travel*) outlined in Table 7.

**Table 7:** Categorising multiple barriers to access

Theme	Categories	Codes	
Barriers to accessing services	Patient level	Patient motivation/QOL/distress (37)	“Whether they decide to engage with more input and strategies to reduce it, is another matter”
		Transport & travel (13)	“The location of the clinic is not always easily accessible to patients particularly given the frequency of sessions required”
	Service level	Lack of services/capacity/out of area (28)	“We do not have any specific lymphoedema treatment available to access. It’s hugely needed”
		Waiting lists (12)	“Waiting lists at Hospice”
		Need for early intervention (4)	“We used to have problems with the head and neck team not referring their patients to our lymphoedema service. Even though we are in the same trust. But having spoken at their team meeting we are now getting the referrals much earlier”
	Referrer level	Cancer status/timing of input (45)	“Where they are in treatment, have to ensure we are not trying to reduce the inflammatory response from radiotherapy rather than lymphoedema”
		Contraindications/cautions/severity (16)	“Episodes of recurrent cellulitis”
		Clinician skills in recognising HNL (6)	“Some medics may be slow to recognise it”

## Discussion

This survey found that although most respondents were able to refer to a HNL service, findings suggest fewer patients (0–25% of their caseloads) are referred for HNL treatment than the incidence suggests in the literature (up to 90%) [2]. This highlights a significant gap in MDT members recognising HNL and referring to services for the provision of HNL treatment. These findings are consistent with current evidence reporting a lack of a robust single referral process or standardised clinical pathway [9]. Results suggest a need for improved education of HNC teams and clearer referral pathways, in particular recognising HNL symptoms after the routine oncology follow-up period has ended, when HNL symptom burden is often at its worst as it evolves into ‘late effects’ of irreversible soft tissue changes [15–17]. Indeed, only four survey respondents specifically mentioned the need for prompt referrals given early intervention has better treatment outcomes [17–19]. The impact of delayed HNL intervention is a concern given that lymphoedema is a chronic and progressive problem, leading to poorer functional outcomes if untreated [20].

The timing of referrals across HNL services was inconsistent. There was also disparity on the number of and rationale for follow-up appointments across services. Prompt referrals were reported by survey participants to be delayed by the need for consultant approval or certain clinicians to assess first. Most treating clinicians cited uniformly accepting HNL referrals from all HNC MDT members, with only a third of services accepting self-referrals exposing an unmet need for self-directed patient access during and after the duration of HNC treatment. Previous patient research reported that having contact details and open access to a HNL specialist would be of value [6]. Consideration of this issue is important when service planning.

Results suggest a lack of pre-treatment preparation across services for patients who are highly likely to experience HNL. This is a missed opportunity to provide education and preventive strategies [21]. Further research into the impact of pre-treatment education and prevention would be beneficial to investigate the role in reducing/preventing lymphoedema or raising awareness to the need of increased referrals [19]. A third of respondents accepted referrals for patients immediately postoperatively. Given the high risk of developing infection from manual manipulation until healing is complete [20], it is perhaps not surprising that treatment is not widely delivered in the immediate postoperative setting.

The survey found inconsistencies in assessment methods, and the level of knowledge, skills and training of treating clinicians. This significant gap of formally trained lymphoedema clinicians in the UK is perhaps counterintuitive to the relatively high levels of recognising internal and external lymphoedema reported by participants. This is in contrast with health care professional research interviews reporting lower levels of confidence recognising HNL [9]. However, survey results related to levels of training are undoubtedly limited by a bias towards nurses and speech and language therapist responders which is not capturing the knowledge, skills and training of occupational therapists and physiotherapists who form a large proportion of clinicians in practice.

Recent systematic reviews have suggested that ‘gold standard’ assessment of HNL should include presence/absence of HNL, severity, physical and patient impact. These assessments should be conducted regularly to evaluate progression and response to treatment [7, 21]. Also of note, only 31% of clinicians used a quality-of-life tool to assess patient impact of HNL. Reasons for this are unknown, although a specific HNL QOL assessment tool has recently become available [22]. Given HNL has a devastating impact on patient’s psychosocial wellbeing [5], this is a missed opportunity to capture this information with the use of QOL patient reported outcome measures to include as a standard of care.

Less than half of respondents reported using a standardised assessment tool and few used a formal rating scale for volume and texture of lymphoedema. The survey results demonstrated a lack of access to and awareness of objective measures of assessment, limited access to high-tech equipment (e.g. ICG imaging and Ultrasound) and appropriately skilled staff with experience of using these methods. Despite respondents reporting that almost all of their patients exhibited both external and internal lymphoedema, assessment of internal structures requires instrumentation such as fluoroscopic imaging or nasendoscopy conducted by a trained individual—which may be unavailable in community settings, exposing an inequity in accessing objective methods of assessing internal lymphoedema.

Research into the efficacy of HNL treatments remains limited [8, 23]. Complete decongestive therapy (CDT) combining compression, manual lymphatic drainage (MLD), skincare and exercise, along with the use of Kinesiotape, were the most common interventions reported by respondents in the survey which is consistent with the standard of care found in current research [8, 23]. Availability of compression specifically was reportedly restricted by service budgets consistent with inequitable service provision found in other surveys. For example, some compression garments are unavailable on prescription and may require patients to self-fund. A small number of services offered photobiomodulation (delivery of infra-red waves to stimulate cellular regeneration). Photobiomodulation equipment is costly and requires further training to deliver this as an intervention. Currently, there is limited evidence for its effectiveness in treating HNL lymphoedema which may explain its exclusion from the current standard of care [24].

Awareness of contraindications to treatment were varied. Of concern is the low numbers reporting serious adverse indicators such as cellulitis which is a common sequalae of lymphoedema [17], leading to sepsis in some instances, and superior vena cava obstruction (SVCO) which is a palliative emergency in patients with advanced disease. Awareness of how SCVO presents symptomatically is crucial to lymphoedema clinicians working within the head and neck area to ensure it is not mistaken for rebound of lymphoedema as this requires immediate escalation for emergency care.

Self-management of lymphoedema treatment was routinely prescribed by respondents which may be more efficient and cost-effective for services [9]. There is a growing body of work investigating why adherence to self-management strategies is poor overall [9, 25]. A more in-depth understanding of what promotes patients’ long-term behaviour change to improve adherence and motivation to HNL self-management techniques would be beneficial within the context of service planning and delivery [9, 25]. Further areas of study to determine consistent standard of care of assessment and management of HNL, including the impact of treatment on swallowing outcomes, are warranted. The results of this study can serve as a reference for conversations at a local level when service planning.

## Limitations

This is the first survey to identify and characterise HNL services in the UK and had a large response rate. However, the findings can only be used as a guide because not all services participated, and the possibility of multiple responses from the same centre, along with a higher proportion of respondents in the West Midlands, may not be representative of the population. Relatively few physiotherapists and occupational therapists responded to the survey compared with previous published surveys of practice [10–14].

## Conclusions

 There are multiple barriers to accessing HNL services, at the patient, referrer and service level. This survey highlights disparity in assessing and treating HNL across the UK. There is a lack of consistency in assessment methods dependant on workforce skills and access to equipment. Internal lymphoedema and QOL measures are not routinely used in practice. The most commonly reported treatments are CDT and Kinesiotape which is consistent with current evidence. Promotion and awareness of the condition, improved workforce skill set, implementation of self-management and multi-disciplinary coordination of services are required.

## Supplementary Information

Below is the link to the electronic supplementary material.

ESM 1 Supplementary Material 1 (DOCX 37.0 KB)

## Data Availability

The data that support the findings of this study are available from the corresponding author upon reasonable request. Data are located in controlled access data storage at University Hospitals Coventry & Warwickshire.
