# Supplementary material for: Head and neck lymphoedema service provision in the UK: a survey of practice
Source: Support Care Cancer. 2025 Nov 27;33(12):1142. doi: 10.1007/s00520-025-10107-6 (PMC12660445; doi:10.1007/s00520-025-10107-6)
Supplement: Supplementary file 1 — Supplementary Material 1 (DOCX 37.0 KB) [file 520_2025_10107_MOESM1_ESM.docx]

Hi Everyone

I'd be eternally grateful if you can spare 10 minutes to complete the survey below. It's part of my research looking into what services are available across the UK for patient’s who present with head and neck lymphoedema after treatment for a head and neck cancer. This survey closes on 31.03.2024 and is open to all health professionals working in HNC. Let me know if there are any issues with the link ([alison.smith3@uhcw.nhs.uk](mailto:alison.smith3@uhcw.nhs.uk)).

I am looking for responses clinicians from the following professional groups within an Inpatient, Outpatient or Hospice setting

• Physiotherapist

• Head and Neck Cancer Clinical Nurse Specialist

• Speech and Language Therapist

• Occupational Therapist

• Dietitian

• Head and Neck Cancer Surgeon

• Head and Neck Cancer Oncologist

• Head and Neck Cancer Radiographer

• Lymphoedema Nurse

[LINK](https://newcastle.onlinesurveys.ac.uk/a-survey-exploring-current-practice-among-uk-head-and-neck-2) FOR SURVEY

**Head and Neck Lymphoedema Service Provision in the UK: A Survey of Practice**

Background

The extent of head and neck lymphoedema services across the UK are not known and are thought to vary across the country in terms of how patients are able to access the services and what treatments are available. This survey is designed to investigate if there are any gaps in service that limit a patient’s access, or if the services offered across the country are equal.

Invitation and Brief Summary

This survey is designed to help get a better understanding of what services and treatments are available within the UK for patients with head and neck lymphoedema following surgery, chemotherapy and/or radiotherapy treatment for head and neck cancer. We are seeking responses from health professionals who work with head and neck cancer patients within the UK. The survey is being conducted by Alison Smith, NIHR Pre- Doctoral Fellow.

What Would Taking Part Involve?

This survey is divided up into two sections. Part A asks some brief questions about your role and what head and neck lymphoedema services are available. Part B asks about resources and skills within these services.

Please take the time to decide which answer best suits your experience for each statement and select accordingly. At the end there is a comments box for any additional information you think is important, not asked within this survey. This should take approximately 3-4 minutes to complete Part A and approximately a further 5-10 minutes to complete Part B if you are a professional who *delivers* HNL treatment.

Please only fill the survey out once if received from different sources. All responses will be reported anonymously, although geographical location will be collated to identify service provision across the UK. Personal information will be kept private, confidential and secure. Email addresses will be removed from the data source. Any potentially identifiable information given in the open text boxes will be anonymised for analysis. Anonymised data will be held on a password protected IT system for two years.

What are the Possible Benefits of Taking Part?

Your feedback will help inform the next steps in establishing a level of service provision for these patients and highlighting any disparity that may exist. No identifiable information will be shared. Under the Data Protection Act, you can at any time ask for access to the information you provide and can also request the destruction of that information. Under these circumstances, in order to retrieve your data, we require your completion receipt number, provided at the end of the survey. This survey has been approved by University of Liverpool Ethics Committee.

If you would like to receive a report of the findings, please contact Alison Smith, NIHR Pre-Doctoral Fellow on the email address below.

If there is a problem with completing the survey or you want more information, please feel free to contact Alison Smith, NIHR Pre-Doctoral Fellow [alison.smith3@uhcw.nhs.uk](mailto:alison.smith3@uhcw.nhs.uk) who will try to help. If you remain unhappy or have a complaint which you feel you cannot come to us with then you should contact the Research Governance Officer at ethics@liv.ac.uk. When contacting the Research Governance Officer, please provide details of the name or description of the study, the researcher(s) involved, and the details of the complaint you wish to make.

Please indicate you are informed about the research and would like to participate by completing the following:

1. I confirm that I have read this information sheet dated 10.11.23 (Version 2) for the above survey. I have had the opportunity to consider the information, ask questions and have had these answered satisfactorily.

[Grab your reader’s attention with a great quote from the document or use this space to emphasize a key point. To place this text box anywhere on the page, just drag it.]

2. I understand that my participation is voluntary and that I am free to withdraw at any time without giving any reason, without my legal rights being affected.

[Grab your reader’s attention with a great quote from the document or use this space to emphasize a key point. To place this text box anywhere on the page, just drag it.]

3. I understand that the information collected about me will be used to support other research in the future, and may be shared anonymously with other researchers.

[Grab your reader’s attention with a great quote from the document or use this space to emphasize a key point. To place this text box anywhere on the page, just drag it.]

4. I agree to take part in the above survey.

[Grab your reader’s attention with a great quote from the document or use this space to emphasize a key point. To place this text box anywhere on the page, just drag it.]

*Many thanks for taking the time to complete this survey.*

**PART A: Clinician Demographics:**

1. What is your profession?
   - (drop down)
   - Physiotherapist
   - HNC Clinical Nurse Specialist
   - Speech and Language Therapist
   - Occupational Therapist
   - Dietitian
   - HNC Surgeon
   - HNC Oncologist
   - HNC Radiographer
   - Lymphoedema Nurse
   - Other (free text)
2. What clinical setting do you work in?
   - (Select all that apply)
   - Inpatient
   - Outpatient
   - Hospice
3. What geographical area do you work in?
   - (drop down)
   - Scotland
   - Northern Ireland
   - Wales
   - North East and Yorkshire
   - East England
   - London
   - Midlands
   - North West England
   - South East England
   - South West England
4. Which HNC treatment centre do the majority of your caseload attend?
   - (free text)
   - I’d rather not say
5. How comfortable do you feel recognising external head and neck lymphoedema?
   - Sliding Scale 1-10
6. How comfortable do you feel recognising internal head and neck lymphoedema?
   - Sliding Scale 1-10
7. What factors do you consider when deciding whether a HNC patient would benefit from HNL treatment?
   - (Free Text) eep
8. Can HNC patients in your service access treatment for HNL?
   - (Drop Down)
   - Yes (will open further questions from 8 onwards)
   - No (thank you for your time in completing this survey)
9. What setting are HNL services provided in your area?
   - (Select all that apply)
   - Inhouse Inpatient
   - Inhouse Outpatient
   - Local NHS Lymphoedema Clinic
   - Regional NHS Lymphoedema Clinic
   - Hospice
   - Private Practitioner/Clinic
10. What barriers to referring HNL patients do you experience if any?
    - (free text)
11. Is surgical or oncological approval required for referring to HNL services?
    - (Drop Down)
    - Yes
    - No
    - Don’t know
12. What % of your HNC caseload do you refer for HNL approximately per year?
    - (Drop Down)
    - Unknown
    - 0-25%
    - 25-49%
    - 50-74%
    - 75-100%

**PART B: Service Provision Provider**

**The following section should be completed if you are clinician that *provides* HNL assessment and treatment.**

1. Who do you accept referrals from?
   - (Select all that apply)
   - Oncologist
   - Head & Neck Surgeon
   - Patient Self-Referral
   - Speech and Language Therapist
   - Occupational Therapist
   - Physiotherapist
   - H&N Clinical Nurse Specialist
   - Palliative Clinical Nurse Specialist
   - (any other profession not included in this list, please elaborate)
2. Which profession(s) will see patients for HNL assessment and treatment in your pathway?
   - Registered Nurse (without formal certified lymphoedema training, CLT certification)
   - Lymphoedema Nurse (with formal certified lymphoedema training, CLT certification)
   - Speech & Language Therapist
   - Physiotherapist
   - Clinical Nurse Specialist
   - Occupational Therapist
   - Any of the above professions with CLT accredited training (free text)
3. At what point of a HNC pathway do you see patient’s for assessment and/or treatment of HNL
   - (Select all that apply)
   - Pre-Surgery (education and preventative)
   - Immediately post operative period (whilst still inpatient)
   - Post Surgery (as outpatient)
   - Pre chemotherapy or radiotherapy
   - During chemotherapy or radiotherapy
   - Post chemotherapy or radiotherapy
   - Late radiation effects
   - Palliative
4. Do you offer the following in your service?
   - (Select all that apply)
   - Initial assessment
   - HNL Treatment clinician delivered
   - HNL Treatment taught home programme for self treatment
   - Direct Follow up
     1. (free text) number of follow ups offered
5. How confident do you feel assessing HNL patients?
   - (Sliding Scale 1-10)
6. How confident do you feel treating HNL patients?
   - (Sliding Scale 1-10)
7. What training have you received relating to HNL?
   - (Select all that apply)
   - Not received any training
   - External certification course (120 hours Certified Lymphoedema Therapist registered)
   - Peer learning
   - In house competency framework?
   - Other external course (not required 120 hours for Certified Lymphoedema Therapist certification)
     1. Please elaborate (free text)
   - None of the above

1. Do you use any standardised assessment tools/protocols when assessing HNL?
   - (Drop down)
   - Yes
   - No
   - Elaborate which tools/protocols (free text)
2. Does your baseline assessment include any of the following:
   - (Select all that apply)
   - Palpation
   - Tape measurements
   - TDC eg Moisture Meter
   - Bio-impedance eg Skin Fibrometer
   - Ultrasound imaging
   - 3D imaging
   - Intra-Oral examination
   - Any other methods of assessment not included in this list (free text)
3. Do you use a formal rating scale for volume/texture of HNL?
   - (Drop Down)
   - Yes
   - No
   - If so, which scale do you use? (free text)
4. Do you use any Quality of Life questionnaires in relation to HNL?
   - (Drop Down)
   - Yes
     1. If yes, which ones? (free text)
   - No
5. What contraindications do you consider as part of your assessment?

- (Free text)

1. Does your HNL intervention include any of the following:
   - (Select all that apply)
   - Manual Lymphatic Drainage (MLD)
   - Self Lymphatic Drainage (SLD)
   - Compression
     1. If compression, what types of garment (free text)
   - Skincare
   - Neck exercises
   - Facial exercises
   - Swallow exercises
   - Hereford Collar
   - Kinesiotape
   - Low level laser
   - Flexitouch, garments
   - Intra-oral drainage clinician led
   - Intra-oral drainage SLD
   - Other (free text)

Please add any other comments on head and neck lymphoedema provision in the box below

If you might be interested in participating in further research on HNL service provision or treatment, please leave your email address ………………………………

Thank you for your participation.
